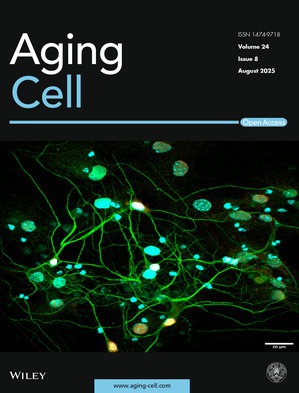# Featured Cover

**DOI:** 10.1111/acel.70200

**Published:** 2025-08-12

**Authors:** Jonathan Plessis‐Belair, Taylor Russo, Markus Riessland, Roger B. Sher

## Abstract

Cover legend: The cover image is based on the article *Nuclear Import Defects Drive Cell Cycle Dysregulation in Neurodegeneration* by Jonathan Plessis‐Belair et al., https://doi.org/10.1111/acel.70091.